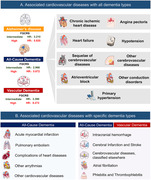# Longitudinal associations of cardiovascular health and vascular events with incident dementia

**DOI:** 10.1002/alz.084314

**Published:** 2025-01-09

**Authors:** Ya‐nan Ou, Lan Tan, Jin‐Tai Yu

**Affiliations:** ^1^ Qingdao Municipal Hospital, Qingdao University, Qingdao China; ^2^ Qingdao Municipal hospital, Qingdao university, Qingdao, Shandong China; ^3^ National Center for Neurological Disorders, Shanghai, Shanghai China; ^4^ Huashan Hospital, Fudan University, Shanghai, Shanghai China; ^5^ Huashan hospital, Fudan University, Shandong China

## Abstract

**Background:**

Evidence supporting cardiovascular diseases could increase the risk of dementia remains fragmented. A comprehensive study to illuminate the distinctive associations across different dementia types is still lacking. This study is sought to: 1) determine the clinical validity of Framingham General Cardiovascular Risk Score (FGCRS) for dementia assessment; 2) examine the associations between cardiovascular diseases and the risk of dementia.

**Method:**

A total of 432079 dementia‐free individuals at baseline from UK Biobank were included. Score points of FGCRS were assigned to corresponding risk factors, including age, sex, smoking status, blood pressure measurements, medication for hypertension, diabetes mellitus, total cholesterol, high‐density lipoprotein cholesterol to compose a rating scale. Participants were further categorized into groups of low, intermediate and high. The date and source of cardiovascular traits via self‐report, primary care, hospital in‐patient admission and death registry were collected under the circulatory system disorders category of the UK Biobank. Multivariable Cox proportional hazard models were used to investigate the prospective associations for FGCRS and a series of cardiovascular diseases with all‐cause dementia (ACD) and its major component, Alzheimer’s disease (AD) and vascular dementia (VaD).

**Result:**

During a median follow‐up of 110.1 months, 4711 individuals were diagnosed with dementia. FGCRS was associated with increased risks across the dementia spectrum. In stratification analysis, high‐risk groups have demonstrated the greatest dementia burdens, particularly to VaD. Over 74 traits, 9 adverse associations, such as chronic ischemic heart disease (ACD: HR = 1.354; AD: HR = 1.269; VaD: HR = 1.768), atrioventricular block (ACD: HR = 1.562; AD: HR = 1.556; VaD: HR = 2.069), heart failure (ACD: HR = 1.639; AD: HR = 1.543; VaD: HR = 2.141), and hypotension (ACD: HR = 2.912; AD: HR = 2.361; VaD: HR = 3.315) were observed. Several distinctions were also found, which atrial fibrillation, and cerebral infarction, and hemorrhage only associated with greater risks of ACD and VaD.

**Conclusion:**

By identifying distinctive associations between cardiovascular diseases and dementia, this study has established a comprehensive “mapping” that may untangle the long‐standing discrepancy. FGCRS has demonstrated its predictivity beyond cardiovascular diseases burdens, suggesting potential opportunities for implantation.